# Comparison of the Direct Effects of Human Adipose- and Bone-Marrow-Derived Stem Cells on Postischemic Cardiomyoblasts in an *In Vitro* Simulated Ischemia-Reperfusion Model

**DOI:** 10.1155/2013/178346

**Published:** 2013-06-19

**Authors:** Mónika Szepes, Zsolt Benkő, Attila Cselenyák, Kai Michael Kompisch, Udo Schumacher, Zsombor Lacza, Levente Kiss

**Affiliations:** ^1^Institute of Human Physiology and Clinical Experimental Research, Semmelweis University, Tűzoltó Utca 37-47, Budapest 1094, Hungary; ^2^Department of Anatomy and Experimental Morphology, Center for Experimental Medicine, University Hospital Hamburg-Eppendorf, Martinistraße 52, 20246 Hamburg, Germany

## Abstract

Regenerative therapies hold a promising and exciting future for the cure of yet untreatable diseases, and mesenchymal stem cells are in the forefront of this approach. However, the relative efficacy and the mechanism of action of different types of mesenchymal stem cells are still incompletely understood. We aimed to evaluate the effects of human adipose- (hASC) and bone-marrow-derived stem cells (hBMSCs) and adipose-derived stem cell conditioned media (ACM) on the viability of cardiomyoblasts in an *in vitro* ischemia-reperfusion (I-R) model. Flow cytometric viability analysis revealed that both cell treatments led to similarly increased percentages of living cells, while treatment with ACM did not (I-R model: 12.13 ± 0.75%; hASC: 24.66 ± 2.49%; hBMSC: 25.41 ± 1.99%; ACM: 13.94 ± 1.44%). Metabolic activity measurement (I-R model: 0.065 ± 0.033; hASC: 0.652 ± 0.089; hBMSC: 0.607 ± 0.059; ACM: 0.225 ± 0.013; arbitrary units) and lactate dehydrogenase assay (I-R model: 0.225 ± 0.006; hASC: 0.148 ± 0.005; hBMSC: 0.146 ± 0.004; ACM: 0.208 ± 0.009; arbitrary units) confirmed the flow cytometric results while also indicated a slight beneficial effect of ACM. Our results highlight that mesenchymal stem cells have the same efficacy when used directly on postischemic cells, and differences found between them in preclinical and clinical investigations are rather related to other possible causes such as their immunomodulatory or angiogenic properties.

## 1. Introduction

Regenerative therapies are representing a relatively new possibility for the treatment of diseases where functional tissue is lost. This approach is aiming to restore organ functionality either by enhancing the resident stem cell population or with substituting the damaged tissue with added cells. Various cell types—such as embryonic, induced pluripotent and adult stem cells—are used to this aim each with its respective ethical, oncological, or immunological advantages and disadvantages [1–4], but data from clinical trials are mostly available from adult stem cells, namely, bone-marrow-derived stem cells (BMSCs) and adipose-derived stem cells (ASCs) [5]. Adipose-derived stem cells have lately become an attractive pool for autologous adult stem cells because of their relatively easy harvest from patients via minimally invasive liposuction [6, 7]. The use of these cells showed promising results and sometimes great success in various situations, such as in articular cartilage regeneration [8], musculoskeletal tissue repair [9–11], and the treatment of chronic, nonhealing wounds [12]. Considering cardiovascular applications, several reports indicated a consistent and significant benefit from cell transplantation after myocardial infarction in *in vivo* animal models [13–19]. Still, the clinical trials using adult stem cell therapy in acute myocardial infarction showed significant but only modest improvements [20–22], and the relative efficacy of the different types of mesenchymal stem cells is still incompletely understood [23, 24]. In this regard, Mazo et al. showed that the transplantation of adipose-derived cells in chronic infarct provided a better left ventricular heart function, less fibrosis, and increased angiogenesis compared to bone-marrow-derived stem cells [25]. Recently, Rasmussen et al. confirmed these data using hypoxically preconditioned adipose- and bone-marrow-derived stem cells from the same patient [26]. Thus, it seems that adipose-derived stem cells are superior to mesenchymal stem cells of other origin. However, no information is provided in these papers on the direct effects of these cells on the postischemic cells. Furthermore, the exact mechanism of action of these cells is also unclear. Initial studies emphasized the role of cell fusion and differentiation as the potentially most important mechanisms of actions [27, 28], but subsequent studies questioned their importance in the beneficial effects [29, 30]. Interest, therefore, switched towards paracrine factors involving proangiogenic, antiapoptotic and anti-inflammatory pathways [31–34]. The importance of the various paracrine effects is also emphasized by the fact that improvements were found in experimental models in spite of the very limited survival of the donor cells in the hostile environment of a damaged tissue [35, 36]. Therefore, in the present study we aimed to evaluate the direct effects of human adipose- and bone-marrow-derived stem cells in a reductionist model of ischemia-reperfusion. Furthermore, we wanted to investigate if mesenchymal stem cells had any direct paracrine effect on the postischemic cells.

## 2. Methods

### 2.1. Cell Lines and Conditioned Media

*H9c2 rat cardiomyoblast* cell line was purchased from ATCC (Wesel, Germany). Cells were cultured in high-glucose (4.5 g/L) DMEM containing 10% fetal bovine serum, 4 mM L-glutamine, 100 U/mL penicillin, and 100 *μ*g/mL streptomycin at 37°C in a humidified atmosphere of 5% CO_2_. Cell culture media was replaced 2 times a week, and cells were passaged once they reached 70–80% confluence.

*Human adipose-derived stem cells* (hASCs) were isolated from liposuction samples of healthy female donors aged 22–50 years (36.4 ± 4.5 years, *n* = 5) who underwent elective cosmetic liposuction after informed consent. The isolation of hASCs from liposuction samples was performed according to an established protocol [37, 38]. Briefly, lipoaspirates were washed extensively with phosphate buffered saline (PBS) and then incubated with 0.075% collagenase at 37°C for 30 minutes. Enzyme activity was neutralized using Dulbecco's modified Eagle's medium (DMEM; Gibco/Invitrogen, Carlsbad, CA, USA) supplemented with 10% heat-inactivated fetal calf serum (FCS), 100 U/mL penicillin, 100 *μ*g/mL streptomycin (all Gibco/Invitrogen), and 100 U/mL nystatin (Sigma-Aldrich, St. Louis, MO, USA). Samples were centrifuged at 1500 rpm for 10 minutes, and the resulting cell pellet was plated in 75 cm^2^-culture flasks (Sarstedt Inc., Newton, NC, USA). Cells were cultured in a 37°C humidified 5% CO_2_ atmosphere. Nonadherent cells were removed after 24 hours. Cells were grown in antimycotic culture medium for 7 days, and culture medium was changed every 2 to 3 days. After that period, hASCs were cultured in low-glucose (1.0 g/L) DMEM containing 10% fetal calf serum, 4 mM L-glutamine, 100 U/mL penicillin, and 100 *μ*g/mL streptomycin at 37°C in a humidified atmosphere of 5% CO_2_. Cell culture media was replaced 2 times a week, and cells were passaged once they reached 70–80% confluence. Cryopreservation was performed on hASCs prior to the experiments, and the revitalized cells were used in the experiments. Passage 1 cells were trypsinized and centrifuged at 1500 rpm for 10 min. Cell pellets were resuspended in CryoSafe medium (c. c. pro GmbH, Neustadt, Germany), aliquoted to cryotubes (Nalge Nunc, Roskilde, Denmark) as 1 mL samples and were stored for 40 minutes at −25°C and then transferred to −80°C for 24 hours followed by final cryopreservation in liquid nitrogen. Human adipose-derived stem cells were characterized by mesenchymal (CD90, CD105, and the stem cell antigen 1 (Sca-1) homolog CD59) and hematopoietic (CD34, CD45) markers with flow cytometry in order to confirm their lineage.

*Human bone-marrow-derived stem cells* (hBMSCs) were isolated from samples gathered from young patients (aged 2–20) during standard orthopedic surgical procedures with the informed consent of the patients or their parents under approved ethical guidelines set by the Ethical Committee of the Hungarian Medical Research Council. All procedures were approved by the Ethical Committee of Semmelweis University. Only such tissues were used that otherwise would have been discarded. The bone marrow was flushed into T75 flasks and diluted with low-glucose (1.0 g/L) DMEM culture medium containing 10% FCS, 100 U/mL penicillin, 100 *μ*g/mL streptomycin, and 4 mM L-glutamine. The flasks were incubated at 37°C in fully humidified atmosphere of 5% CO_2_ and 95% air for 3 days. After the incubation period, the hBMSCs adhered to the surface of the flasks and the remaining components of bone marrow were eliminated by washing with PBS. The used hBMSCs were cultured in the same conditions as hASCs. Human bone-marrow-derived stem cells were characterized by mesenchymal (CD73, CD90, CD105, and CD166) and hematopoietic (CD34, CD45) markers with flow cytometry in order to confirm their lineage. Characterization was performed on cells cultured under standard culture conditions and growing as monolayers while displaying constant cell proliferation rates over the entire culture period.

For preparing *conditioned media* (ACM) adipose-derived stem cells were used because their proliferation capabilities are much better compared to hBMSCs which helped to achieve the highest possible concentrations of paracrine molecules in the medium in the given period of time [39]. Human ASCs were seeded at 10.000 cells/cm^2^ in 100 mm Petri dishes using 8 mL low-glucose (1.0 g/L) DMEM culture medium containing 10% FCS, 100 U/mL penicillin, 100 *μ*g/mL streptomycin, and 4 mM L-glutamine. The dishes were incubated at 37°C in fully humidified atmosphere of 5% CO_2_ and 95% air, and cell-free supernatants were collected for further experimental use after 48 hours. 

### 2.2. *In Vitro* Ischemia-Reperfusion Model

**Figure 1:**
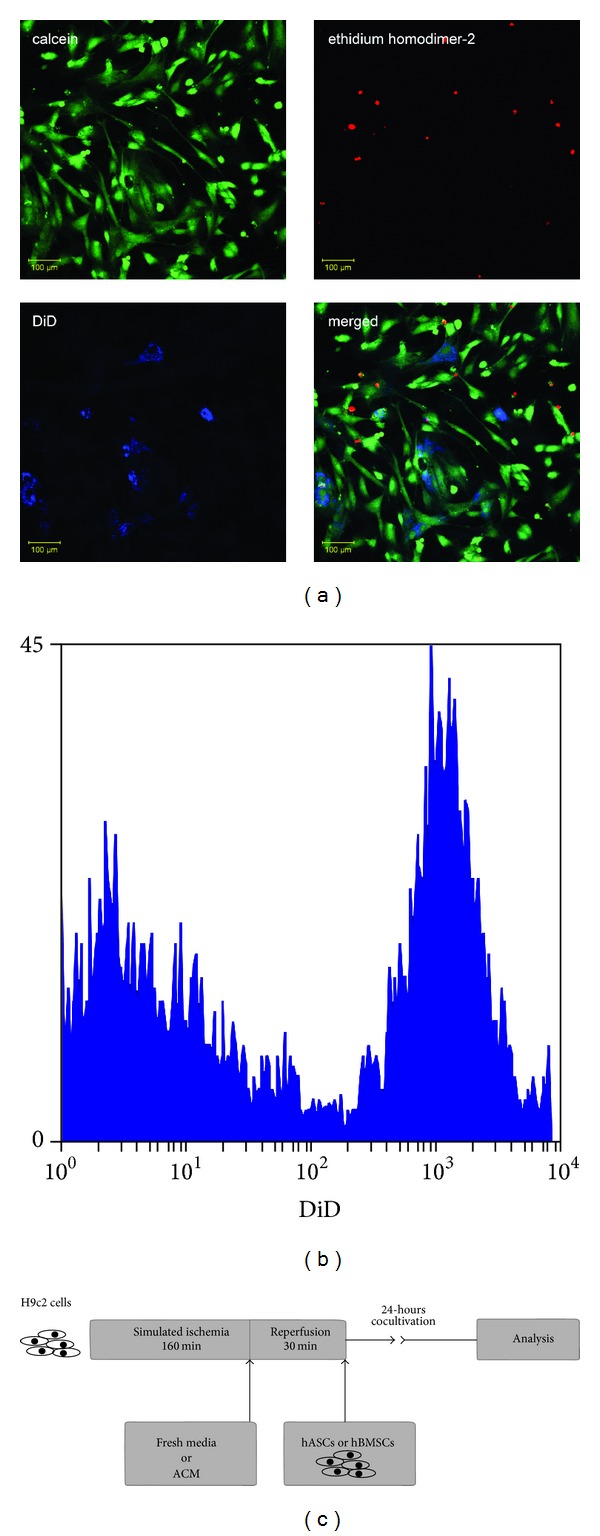
Ischemia-reperfusion model. (a) Representative fluorescent microscopic picture showing H9c2 cells injured with our ischemia-reperfusion model and treated with Vybrant DiD (ex/em: 644/665 nm, blue) labeled cells. The cytoplasm of the living cells is stained with calcein-AM (ex/em: 494/517 nm, green), the nuclei of the necrotic cells are ethidium homodimer-2 stained (ex/em: 536/624 nm, red). (b) Flow cytometric histogram on the distinction between stem cells and H9c2 cells based on DiD staining. (c) Schematic representation of the experimental protocol.

Ischemia-reperfusion was simulated *in vitro* by oxygen and glucose deprivation as described previously in our earlier publications [40–42]. Briefly, 30.000/well H9c2 cells in 12-well plates were incubated in glucose-free DMEM in an atmosphere of 0.5% O_2_ and 99.5% N_2_ for 160 minutes. This procedure was performed on an established incubation system (PeCon, Erbach-Bach, Germany). After incubation, the cells were reoxygenated and glucose was provided by immediate replacement of the media with fresh high-glucose DMEM, and the cells were kept in standard cell culture conditions till further experimental actions. Representative fluorescence microscopy pictures were taken to follow the cell viability during the model using a Zeiss LSM 510 META (Carl Zeiss, Jena, Germany). We used calcein-AM (ex/em: 494/517 nm, Invitrogen, Carlsbad, CA, USA) and ethidium homodimer-2 (ex/em: 536/624 nm, Invitrogen, Carlsbad, CA, USA) labeling to mark live/dead cells. Added mesenchymal stem cells were dyed with Vybrant DiD (ex/em: 644/665 nm, Invitrogen, Carlsbad, CA, USA) (Figure 1(a)).

### 2.3. Experimental Protocol

Four experimental groups were investigated in which postischemic cells received: (1) normal medium (I-R-model); (2) hASC conditioned medium (ACM); (3) hASCs; and (4) a group that received hBMSCs. Cell-treated groups were given 20.000 cells 30 minutes after the reoxygenation, and the added cells were labeled with Vybrant DiD fluorescent membrane dye to enable differentiation from the postischemic cells (Figure 1(b)). Cells were cocultivated for 24 hours in standard cell culture conditions. In the case of ACM group at the end of simulated ischemia, the glucose-free medium was changed to same volume of cell-free hASC conditioned media (Figure 1(c)). 

### 2.4. Cell Viability Measurement with Flow Cytometry

Twenty-four hours after reoxygenation, cells were harvested by trypsinization and resuspended in 500 *μ*L PBS containing 5 nM calcein-AM and 350 nM ethidium-homodimer-2 for flow cytometric analysis [43]. Controls were prepared as follows: for live control, cells were cultured in standard conditions; for dead control, cells were treated with 100 mM H_2_O_2_ for 1 hour immediately before trypsinization. For the measurements, FACSCalibur flow cytometer (Becton Dickinson, Franklin Lakes, NJ, USA) was used and the data was analyzed with the Weasel program (The Walter and Eliza Hall Institute, Parkville, VIC, Australia). Using flow cytometry, we could distinguish the therapeutically given cells from the postischemic cells on the basis of their DiD labeling, and these cells were gated in or out as appropriate for further analysis. 

### 2.5. Metabolic Activity Measurement

For the evaluation of the metabolic activity in the groups, we used the PrestoBlue Cell Viability reagent (Invitrogen, Carlsbad, CA, USA), according to the manufacturer's instructions. Because of the relative low cell numbers used in the experiments, we chose a 24-hour incubation time in 37°C and measured absorbance as instructed.

In the hASC and hBMSC groups to exclude the influence of the added stem cells metabolism, we subtracted the metabolic activity value of 20.000 stem cells from the measured value. The obtained results are compared to the metabolic activity of 30.000 H9c2 cells cultured in standard cell culture conditions (control).

### 2.6. Lactate Dehydrogenase (LDH) Cytotoxicity Assay

The measurement was performed using LDH Cytotoxicity Kit II (PromoCell, Heidelberg, Germany) according to the manufacturer's instructions, with 30-minute incubation period and absorbance measurement at 490 nm. For the LDH measurements, the previously described experimental groups were used 24 hours after ischemia-reperfusion. The LDH enzyme level was determined in the supernatant of 30.000 H9c2 cells cultured in standard conditions (control). The absorbance results were normalized with the total cell number in each sample. 

### 2.7. Statistics

Statistical analysis of data was carried out either with one-way analysis of variance with Newman-Keuls multiple comparison post hoc test or unpaired *t*-test as appropriate. All data are expressed as mean ± SEM. A *P* value of <0.05 was accepted as statistically significant.

## 3. Results 

### 3.1. Characterization of hASCs and hBMSCs

**Figure 2:**
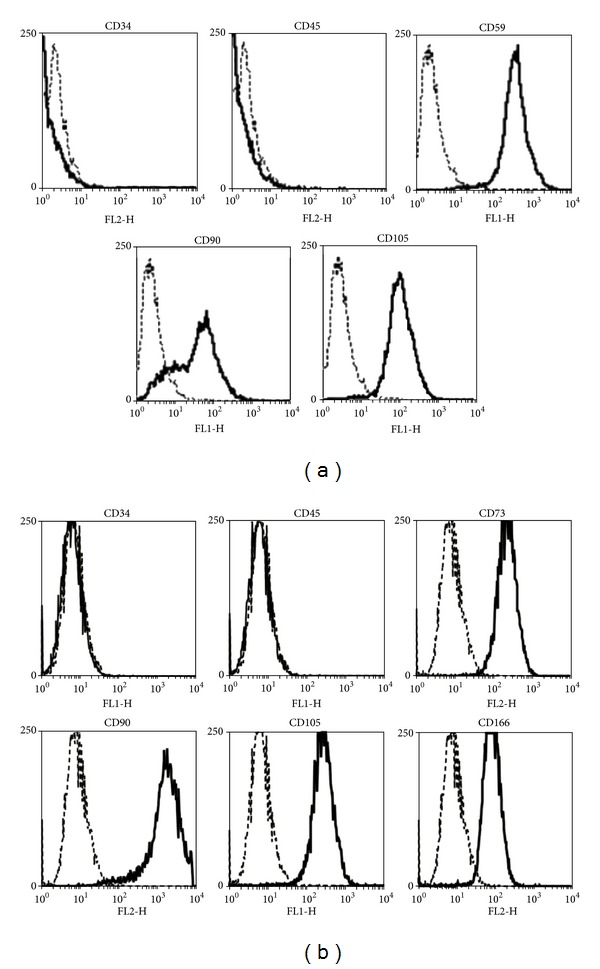
Characterization of adult stem cells. Flow cytometric analysis revealed a CD34^−^, CD45^−^ and CD59^+^, CD90^+^, CD105^+^ pattern for hASCs (a) and a CD34^−^, CD45^−^ and CD73^+^, CD90^+^, CD105^+^, CD 166^+^ pattern for hBMSCs (b). The isotype controls are indicated with dashed lines.

Analyses of cell surface markers by flow cytometry demonstrated that hASCs (Figure 2(a)) were positive for the mesenchymal stromal (stem) cell markers CD90 and CD105 as well as the stem cell antigen 1 (Sca-1) homolog CD59 and were negative for the lymphohaematopoetic markers CD34 and CD45. Flow cytometry analysis of cultured bone-marrow-derived stem cells (Figure 2(b)) exhibited the lack of hematopoietic markers (CD34^−^, CD45^−^), but revealed mesenchymal stem cell lineage specific cell surface markers (CD73^+^, CD90^+^, CD105^+^, and CD166^+^). With respect to cell surface marker expression, our findings were consistent with previous reports [44, 45]. 

### 3.2. Flow Cytometric Viability Analysis

**Figure 3:**
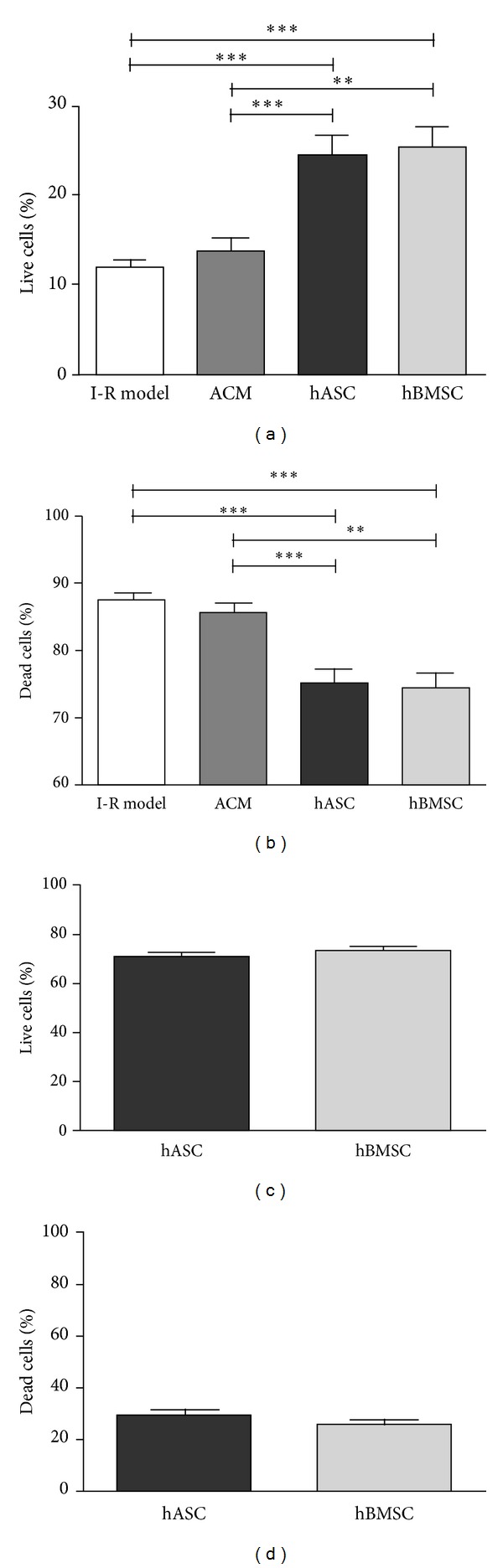
Flow cytometric analysis of the postischemic cells and the therapeutic cells after 24 hours. Flow cytometric cell death analysis of the postischemic cells revealed that cell treatment increased the percentage of live cells (a), while ACM did not (I-R model: 12.13 ± 0.75%; ACM: 13.94 ± 1.44%; hASC: 24.66 ± 2.49%; hBMSC: 25.41 ± 1.99%). (b) The percentage of dead cells decreased when therapeutic cells were added (I-R model: 87.71 ± 0.82%; ACM: 85.75 ± 1.57%; hASC: 75.24 ± 2.49%; hBMSC: 74.62 ± 1.99%). The percentages of live (c) and dead (d) cells among the therapeutically added cells were not significantly different (*n* = 17–31, **P < 0.01, ***P < 0.001).

Our experimental results regarding the postischemic cells showed that without any treatment live cells amounted to 12.13 ± 0.75% after 24 hours. However, the percentage of live cardiomyoblasts after 24 hours was significantly increased both with hASC (24.66 ± 2.49%) and hBMSC (25.41 ± 1.99%) treatments but not with the addition of ACM (13.94 ± 1.44%). There was no significant difference between the cell-treated groups. Cell-treatments led to a significantly increased percentage of live cells compared to ACM treatment as well (Figure 3(a)).

The percentage of the dead cells in the I-R model group was 87.71 ± 0.82%, while this was significantly smaller in the hASC and hBMSC treated groups (hASC: 75.24 ± 2.49%; hBMSC: 74.62 ± 1.99%), but was not statistically different in the ACM treated group (ACM: 85.75 ± 1.57%). There was no significant difference between the hASC- and hBMSC-treated groups (Figure 3(b)). 

Putting the added cells into consideration, we found that most of the stem cells were alive in both the hASC-treated group (70.30 ± 2.35%) and the hBMSC-treated group (73.30 ± 1.92%), and there was no statistically significant difference between the groups (Figure 3(c)). Furthermore, no difference was found considering the dead cell population (hASC: 29.20 ± 2.42%, hBMSC: 25.81 ± 1.89%; Figure 3(d)).

### 3.3. Metabolic Activity Measurement

**Figure 4:**
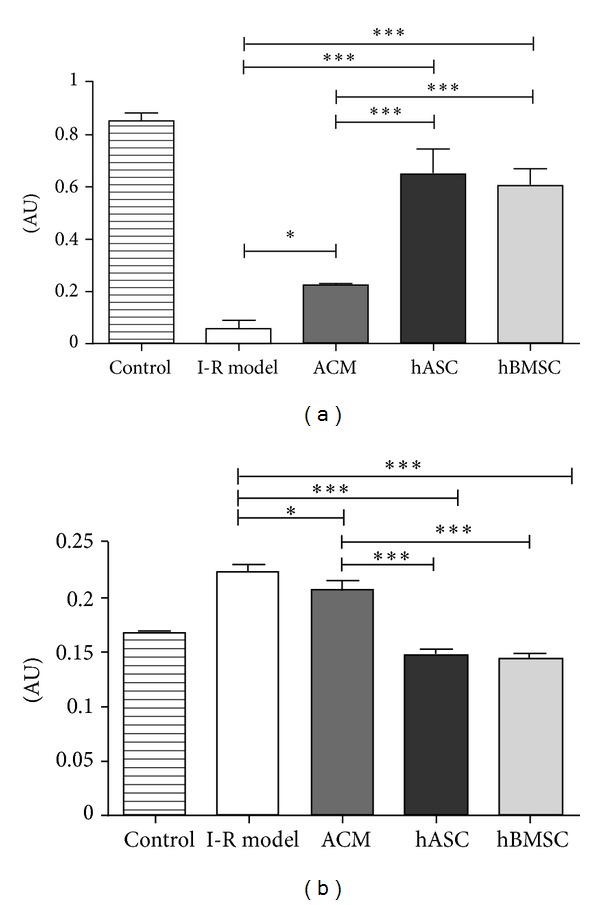
Metabolic activity measurement and LDH assay. (a) The metabolic activity measured 24 hours after the ischemia-reperfusion injury significantly decreased in the cells after the ischemic conditions compared to the control group, but the metabolic activity was enhanced with all the applied treatments (control: 0.858 ± 0.021 AU; I-R model: 0.065 ± 0.033 AU; ACM: 0.225 ± 0.013 AU; hASC: 0.652 ± 0.089 AU; hBMSC: 0.607 ± 0.059 AU; *n* = 3, *P < 0.05, ***P < 0.001). (b) LDH levels in the cell culture supernatant were significantly lower when ACM, hASC, or hBMSC therapy was carried out (I-R model: 0.225 ± 0.006 AU; ACM: 0.208 ± 0.009 AU; hASC: 0.148 ± 0.005 AU; hBMSC: 0.146 ± 0.004 AU; *n* = 3, *P < 0.05, ***P < 0.001). The stem cell treated groups are not significantly different from each other and also not different from the control.

Using the PrestoBlue colorimetric assay, we strengthened our earlier findings with the flow cytometric analysis. The reducing capability of the cells reflecting their viability was significantly higher after treatment with hASCs (0.652 ± 0.089 AU, arbitrary units) and hBMSCs (0.607 ± 0.059 AU) compared to the I-R model (0.065 ± 0.033 AU). Moreover, the treatment with ACM was also able to increase the metabolic activity of the postischemic cells (0.225 ± 0.013 AU). No difference was observed between the beneficial effects of the two different stem cell lines (Figure 4(a)).

### 3.4. Lactate Dehydrogenase Cytotoxicity Assay

Cellular necrosis expressed by LDH release decreased significantly compared to I-R model (0.225 ± 0.006 AU) when the postischemic cells were treated with hASC and hBMSC (hASC: 0.148 ± 0.005 AU; hBMSC: 0.146 ± 0.004 AU). Conditioned media could decrease the LDH levels only very slightly (0.208 ± 0.009 AU). In case of hASC and hBMSC treatments, the necrosis was not significantly different from the control (Figure 4(b)).

## 4. Discussion

We report here that human adipose- and bone-marrow-derived cells directly improve the survival of postischemic cardiomyoblasts in an *in vitro* reductionist model. Metabolic activity measurement and the evaluation of necrosis strengthened the beneficial effect of cell treatment. Importantly, there was no difference in these direct effects between the adipose- and bone-marrow-derived stem cells.

Furthermore, the percentage of live mesenchymal stem cells after 24 hours was the same, so their survival properties are also likely to be similar. These observations are important because many publications indicate a better result with adipose-derived stem cells, but the underlying mechanisms of action are not clearly understood yet, and, to our knowledge, this is the first report on the comparison of the direct effects of adult stem cells on the parenchymal cells of the damaged tissue and on their survival in a standardized situation. Rasmussen et al. have shown that adipose-derived stem cells had preserved cardiac function following myocardial infarction in their animal model while bone-marrow-derived stem cells from the same source had not [26]. They reported that neither of these cell types induced angiogenesis. Thus, based on a recent report [39], they argued that the potential difference between them could be explained by differences in senescence properties of the cells. Others showed in an investigation on spinal cord injury that adipose-derived cells increased angiogenesis more than cells from other mesenchymal sources and expressed higher amounts of VEGF while having similar migration properties to the bone-marrow-derived stem cells [46]. Adipose-derived stem cells were also found to be more effective on cutaneous wounds upregulating fibroblast migration and proliferation [47]. However, this may prove to be problematic in case of myocardial regeneration due to increased possibility of scar formation. Finally, a comparative study indicated ASCs to be a more promising source because of its more favorable immunomodulatory effects [48]. 

We have drawn a few conclusions from our data on the possible mechanisms of actions, also. First, LDH levels decreased to control levels after cell treatment. This means that necrosis was practically blocked by the added cells, indicating that the dead cells in our study were apoptotic cells. This is beneficial as apoptotic cells were shown to be immunoregulatory, and some researchers argue that the main effect of the current cytotherapy is aspecific and is the consequence of this apoptotic pool [36]. However, it must be realized that this possibility does not explain our results on cell viability as our model is completely reductionist and contains no immune cells. Second, the conditioned medium slightly increased metabolic activity and decreased LDH levels; thus, it had an antinecrotic effect. It means that the paracrine cocktail released from mesenchymal stem cells contains substances that act directly on the ailing postischemic cells. The enhanced metabolic activity may relate to slightly better functionality of the surviving cells while the decreased LDH-levels indicate that the postischemic cells are directed from necrosis towards apoptotic cell death because the ACM had no effect on the cell viability in the flow cytometric measurements which is in accordance with our previous work using bone-marrow-derived cells in cell culture inserts [40].

In our study, the ineffectiveness of the stem cell conditioned media versus the stem cell treatment in increasing live cell numbers means either that the cell-cell contact is particularly important in the direct beneficial effect or the reached concentration of paracrine molecules is not high enough for their effect. The importance of cell-cell contact in the actions of therapeutically added cells was highlighted in earlier studies where the mechanism was related to intercellular tubular connections that potentially lead to mitochondrial exchange between the cells [40, 49]. Cell fusion is another phenomenon which can be observed in coculture studies, and in some cases it was also observed in *in vivo* animal studies of stem cell grafting [30, 50, 51]. The possibility of cell fusion in our model was addressed in our first publication and its frequency was found to be extremely low [40]. However, it must be noted that the extent of cell fusion shows extremely high variation among different culture and detection techniques, and it cannot be ruled out that extensive cell fusion is an *in vitro* artifact [28, 52, 53]. Still, recent studies suggest that despite the low frequency cell fusion may exert relevant impact on stem cell programming or reprogramming in the heart [54]. In view of the recent literature, it is more probable that the mechanism is mediated via paracrine factors, but the stem cells have to be induced by the microenvironment or by contact with injured cells to release these beneficial factors in necessary amounts. It is also possible that during the production of the conditioned media the concentration of paracrine factors in the conditioned media did not reach the levels necessary to be effective. No wonder, studies of late started to concentrate the conditioned medium to achieve higher concentrations and found promising results [55]. Our approach raises the possibility that the secreted molecules are effective only in close proximity to the affected cells where their local concentration can reach high levels. It is highly possible that only direct cell-to-cell contact can provide the necessary distance. It is interesting to note, that such “microparacrine” mechanism exists in relation of stem cells in the bone marrow, where the so-called “endosteal niche-stem-cell synapses” are formed [56]. A final, additional concern could be that conditioned media has a predominant role in angiogenesis; thus, it is ought to be ineffective in our reductionist model [26].

The relative role of the observed direct mechanism in the *in vivo* setting is difficult to measure, but it may be quite robust if we consider that we observed a doubling in the number of live cells. However, it must be realized, that in our experimental model the majority of the therapeutically added stem cells survived unlike the *in vivo* situation where most of the injected cells die soon after the transplantation [36]. Thus, the added cells had a prolonged time for exerting their effect. 

At this point, it may be useful to consider some experimental points in our study. Our experimental model was devised to investigate acute effects of therapeutic stem cells on severely damaged cells to give room for the potential effects of stem cells. For this reason, we have set the length of simulated ischemia to a level where only 5–20% of cells survived without any treatment, which reflects the conditions found at the site of the injury in the heart after myocardial infarction. We demonstrated the suitability of this model by detecting significantly elevated levels of oxidative stress and cellular necrosis after the simulation of ischemia-reperfusion in our earlier publication [42]. The effects were analyzed at 24 hours because we wanted to rule out the potential differentiation of the added stem cells, which occurs over longer time periods. 

Some limitations must be accounted for considering our study. In our experiments, we used H9c2 cells which are derived from rat embryonic heart tissue. Obviously, there are differences between these cells and human adult cardiomyocytes, but H9c2 cells are frequently used in studies dealing with reperfusion injuries, and we have ample experience with these cells in our model [57–59]. Furthermore, as our aim was to analyze the direct effects of cells or media on postischemic cells, using H9c2 cultures instead of adult cardiomyocyte cell cultures we could avoid the possibility that inflammatory cells would contaminate the cultures and affect the results. Still, it has to be kept in mind that we used human cells for treatment, but as no immune functions were involved in our model this fact must not had any major effect on our results, and human therapeutic cells are widely used in the literature in animal disease models [60–62]. Also, in our experiments we used an *in vitro* approach to the much more complex issue of stem cell therapy in myocardial infarction, with all the disadvantages and advantages of such model. An *in vitro* transplantation model in a cell culture system cannot mimic the 3-dimensional tissue where cell-to-cell connections are different and it cannot reflect the complex (e.g., immunological) events taking place during and after myocardial infarction. However, a limitation of the *in vivo* models in cell treatment studies is a lack of separation between the direct effect on the treated parenchymal cells and the indirect effect caused by alteration of the environment (e.g., inactivation or reduced migration of leukocytes, angiogenesis, etc.). We believe that our model was appropriate for the scope of our study because it can focus on the direct effects of the added cells on the postischemic cells. The similar benefits achieved with hASC treatments strengthen that hASCs can be an alternative to the most commonly used hBMSCs in the emerging field of cell therapy. Subcutaneous adipose tissue is an attractive source for obtaining autologous mesenchymal stem cells as it can be harvested easily by liposuction which is performed routinely on thousands of people per year. The yield of stem cells per gram of adipose tissue is reported to be superior to that which can be achieved per milliliter of bone marrow [63], and adipose tissue can be harvested safely in much higher quantity. This is important as stem cells constitute only a small portion of cells in bone marrow and their number and differentiation capacity correlate inversely with age [64]. Similarly to hBMSCs, hASCs were shown to be able to differentiate toward osteogenic, adipogenic, myogenic, and chondrogenic lineages [6, 37, 64] and to secrete a host of paracrine factors that can increase angiogenesis and act as antiapoptotic signals [18, 31]. As their direct effects are at least as good as the effects of hBMSCs, our results strengthen the assumption that they constitute a better and more practical source for therapies using adult stem cells. No evidence is available to date, but two Phase I clinical trials have been recently completed to test the safety and feasibility of adipose-derived mesenchymal stem cell treatment in myocardial infarction and in chronic myocardial ischemia (APOLLO, NCT00442806; PRECISE, NCT00426868) [65]. 

## 5. Conclusions

Our results highlight that adipose-derived and bone-marrow-derived stem cell treatments can directly save damaged cardiomyoblasts with the same efficacy. The survival of these cells in the noxious, oxidative environment is also similar. These results may indicate that if these cells arrive to the injury site the resulting direct effect will be similar on the cardiac cells so the observed differences in efficacy found in *in vivo* experiments and in clinical trials may relate to different properties in homing, angiogenesis induction, fibroblast regulation, or immunomodulation.
